# War Wounds Treated by Primary Suture and Delayed Primary Suture

**Published:** 1918-03

**Authors:** G. E. Gask


### WAR WOUNDS TREATED BY PRIMARY SUTURE AND DELAYED PRIMARY SUTURE

By Colonel G. E. GASK, A. M. S.

*Explanatory Introduction.* — On December 27, 1914, one C.C.S. in the 4th Army was set apart for the purpose of investigating the possibilities of Primary and Delayed Primary Suture of Wounds. The work was placed under the direction of Capt. Forbes Frazer, F.R.C.S., R.A.M.C., and the figures quoted in this paper are the results obtained in this hospital up to February 4, 1918. Arrangements were made whereby patients could be kept under observation until their wounds were healed.

*Surgeons.* — Six different surgeons were selected to work, so that the results are not those of one individual, who might be particularly skilful.

*X-Ray.* — Every wound in which there was a possibility of the retention of a foreign body was screened before operation.

*Bacteriology.* — A careful bacteriological examination was made of the wound at the time of operation and a report rendered to the surgeon at the earliest possible moment, particularly as to the presence of streptococci. Cultures both aerobic and anaerobic were made. Each surgeon used three methods of treating the wounds:

*a*) Without antiseptics.

*b*) With B.I.P.P.

*c*) With ether.

*Antiseptics.* — It was left to the discretion of the individual surgeon to choose the method. It is evident that the surgeons have a great desire to put something into the wound. The figures quoted below refer only to completed cases in which a definite result has already been obtained.

| | |
|---|---|
| 1. A) *Number of Primary Sutures* | 123 |
| Successes Complete | 79 |
| — Partial (where absence of skin has prevented complete closure) | 23 |
| | 102 = 82.9 o/o |
| — Failures | 21 = 17.1 o/o |

*Average Interval Between Wound and Operation.*

| | |
|---|---|
| (1) In all cases | 10.3 hours |
| (2) In successful cases | 9.2 — |
| (3) In failures | 10.7 — |

The longest interval between time of wound and operation followed by successful primary suture was 28 hours.

*Comparison of Methods.*

| | | |
|---|---|---|
| (1) Without antiseptics | Successes | 24 |
| | Failures | 1 |
| (2) With B. I. P. P. | Successes | 50 |
| | Failures | 11 |
| (3) With Ether | Successes | 28 |
| | Failures | 9 |

This comparison is of little value on account of the tendency of surgeons to treat only the lighter cases without antiseptics and the more difficult ones — e. g. compound fractures — with B. I. P. P. in which they have the tendency to place reliance.

*Compound Fractures of Bones.*

The following cases have been treated by Primary Suture :

| | | | | |
|---|---|---|---|---|
| Humerus | 3 | Successes | 3 | |
| | | Failures | 0 | |
| Metacarpus | 1 | Successes | 1 | |
| | | Failures | 0 | |
| Femur | 7 | Successes | 2 | 1 treated successfully by delayed primary suture) |
| | | Failures | 5 | |
| Tibia and Fibula | 1 | Successes | 1 | |
| | | Failures | 0 | |
| Tarsus | 1 | Successes | 1 | |
| | | Failures | 0 | |
| Metatarsus | 1 | Successes | 1 | |
| | | Failures | 0 | |

*Causes of Failure of Primary Suture.*

(1) Faulty technique whereby dead tissue is not removed. It is to be noticed that with practice the percentage of successes is rising.

(2) The presence of streptococci in the wound and failure to eliminate them. The numbers will be referred to later under the heading Bacteriology.

B. *Delayed Primary Suture.*

(i. e. after 3-4 days but before the wound granulates freely.)

In this series the wound was excised, left open, and packed with gauze soaked in Flavine 1 in 1000 and covered with jaconet. In some cases Carrel's tubes were left in the dressings and for an injection, Flavine solution was used in order to keep the dressings moist.

Usually on the third day the wound was dressed under anesthesia washed with Flavine and sewed up.

| | |
|---|---|
| Completed cases | 15 |
| Perfect union | 11 |
| Partial union | 3 |
| Failure | 1 |
| | (soft part) |

These cases include five compound fractures.

| | | |
|---|---|---|
| Humerus | 2 | |
| Radius | 1 | |
| Fibula | 1 | (all successful) |
| Astragalus | 1 | |
| | 5 | |

1 amputation of thigh for shattered knee.

1 closure of large wound by flap-siding operation.

All these wounds were closed on the third day with two exceptions in which the closing took place on the fifth day. All were infected as shown by culture, prior to operation. In 13 cases smears were made before secondary suture; in 8 no organisms were seen: in the remaining 5, organisms were still present at time of suture and the wounds healed.

The results of delayed primary suture compare very favorably with those of immediate primary suture, though perhaps the scar is not quite so free from inflammatory reaction.

All cases referred to were kept under observation until healed.

There appears to be little difference in time of convalescence between cases of primary suture and those of delayed primary suture.

It is noticed that : (1) though many of the cases in the above series were severe, no deaths occurred; (2) no bad results were attributable to the early suture ; (3) the comfort and well-being of the patients were noteworthy.

### II. — Indications and contra-indications, inherent in the wound, for primary suture.

*Indications for Primary Suture.*

All wounds, other than very insignificant ones, which can be cleansed completely and mechanically within say 12 to 24 hours after receipt of wound, and which can be retained in bed for a period of say 7 days before being submitted to a journey by train or ambulance car.

(*N. B.*). — In my opinion the above statement may be open to revision as regards lightly wounded men. I think that the time limit before operation might be extended to 24 hours, and I think it possible that a man with a small, superficial wound, completely excised and sutured, might be evacuated without harm. More evidence on these points is desirable.

*Contra-indications for Primary Suture.*

(1) Small, superficial, insignificant wounds requiring no treatment.

(2) Small, clean, perforating bullet wounds.

(3) When patients cannot be retained for 7 days after operation.

(4) Badly shocked patients for whom the long operation necessary for primary suture would constitute a danger to life.

(5) Multiple wounds of great severity for the same reason.

(6) Wounds which the surgeon cannot hope to cleanse mechanically, e. g. — *a*) Wounds exposing or injuring large vessels or nerves ; *b*) large shattering wounds of bones.

(7) Wounds already showing active signs of inflammation, i. e., wounds in which organisms have already penetrated living tissue. In this stage much harm may be done by too free surgery, by exposing fresh planes of tissue to infection.

The number of hours after which this invasion of living tissue occurs varies considerably, the variation being due probably to a number of factors, e. g. loss of blood, amount of infection, cold, state of weather — thus a man with a large wound of the leg, lying in a muddy shell hole would probably become infected earlier than a man lying on dry sand.

Cases included in the above category, which need operation, should be treated as efficiently as opportunity allows and kept for delayed primary or secondary suture.

When accommodation will not allow retention of cases for 7 days the wound should be excised with the same care as for primary suture, dressed aseptically or antiseptically, then sent away to an area where the delayed primary suture can be carried out.

*Operative Technique Employed in Primary Suture.*

1. Preliminary screening of all cases in which there is a possibility of retention of a foreign body and, when necessary, localization.

2. Anesthesia.

3. Preparation of skin, as for civilian operation.

4. Excision of wound.

Asepsis must be as rigid as in civilian work — as far as possible fingers should be kept out of the wound, the necessary manipulations being performed with forceps, knife, scissors and retractors.

The aim of the operation is the removal of every particle of dead or badly damaged tissue, all fragments of metal, débris, portions of cloth, and completely detached splinters of bone.

This is not done " en bloc " as has been suggested.

The excision should proceed systematically, say from one end of the wound to the other, in order to save time and to avoid overlooking any portion.

The work should be as conservative as possible, no more living tissue than is necessary being removed — skin edges are merely trimmed.

In order to obtain complete removal of all dead tissue it is essential to have a good exposure of the whole extent of the wound, necessitating sometimes a long incision : in planning the incision this should be kept in mind; also wherever possible the incision should be in the long axis of the limb.

In the case of entry and exit wounds, both requiring excision, the track may be slit up along its whole length or in the case of limbs the excision may be on the double cone system.

*Closure of the Wound.* — The main principles are :

(1) No cavities should be left capable of filling up with blood or serum.

(2) Surfaces should be approximated with as little tension as possible and skin sliding or flap sliding resorted to when necessary.

(3) Buried stitches are to be avoided when possible.

(4) Suture material should be impermeable, e. g. fishing gut, wire or " Bipped " silk — good results have been obtained with the last.

(5) Drainage tubes are not necessary and probably are even harmful — good drainage may be ensured by a piece of wire (aluminium bronze) inserted in a corner of the wound.

*Technique in Cases of Compound Fracture.* — These are undoubtedly the most difficult to treat and much must yet be done before we can suture grossly shattered bones with confidence.

It seems generally agreed that small, completely loose, splinters should be removed.

There is disagreement on the correct treatment of stellate and splintered but attached portions of bone. Should the splinters be levered apart and curetted or is it sufficient to use a fine brush for the purpose?

It seems clear that a cavity left in a bone, as for instance in the cancellous tissue of the lower end of the femur or upper end of the tibia is a source of danger for primary suture — the cavity fills with fluid, becomes infected and breaks down.

As soon as the necessary apparatus arrives we propose to clean out such a cavity by a mechanical burr, treating it as a dentist does a septic cavity in a tooth. Having cleaned it out, washed it out, and dried it out, we then propose to stop it, using as filling either an autogenous fat or bone graft.

*Note on Coloring Reagent.* — 5 o/o sol. of methylene blue in 20 o/o formalin as recommended by Le Grand. My opinion of this is: that it is certainly of use in distinguishing living from dead tissue, that when learning the methods of excision for primary suture it is a useful aid, but that after a considerable amount of experience its use may be abandoned.

*N. B.* — The operation to obtain primary suture takes longer than the old rough method we have been employing. The extra time taken by the operation is made up a hundred times over by the shortening of convalescence, the saving of time in attendance and nursing, and the cost of dressings.

*Operative Technique for Delayed Primary Suture.* — This is exactly the same as for primary suture, except that the wound is sewn up about three days later, the wound during the interval being kept moist by means of some wet dressing, or B. I. P. P., and most important of all, kept free from fresh infection. If such a wound has to be dressed during the interval, the most vigorous asepsis is essential.

*After-Treatment of Primary or Delayed Primary Suture.* — 1. Rest. This is most important — the cases should not even be dressed unless symptoms arise necessitating examination of the wound. Most of the cases in the above series were not dressed until the 8th or 10th day, when stitches were removed.

2. If the bacteriologist reports the presence of a hemolytic streptococcus, the wound should be opened up at once, all stitches removed and the case reserved for secondary suture. Out of 123 wounds primarily sutured, 8 were infected with hemolytic streptococci; of these 6 were complete failures, 1 was partially successful and 1 (a knee-joint) completely successful.

3. The temperature and pulse rate must be watched. It is common to find a slight rise of temperature following suture; the wounds, no doubt, are infected, but the temperature commonly falls next day. If, however, there is a rise of temperature and pulse the wound should be examined. A wounded part infected with streptococcus is swollen — the skin edges are reddened and edematous and a thin fluid exudes between the stitches. In such cases, stitches should be removed and even if a bacteriologist is not at hand to report on the presence or absence of streptococci a little experience soon shows which wound may be left alone and which must be opened.

4. One of the important points of primary suture is the possibility of early restoration of function. In my opinion active movements may be commenced very early in those cases which are not septic. Movements should be encouraged after a few days *up to the limit of toleration*. Early passive movements I regard with some suspicion and if carried out they should be supervised with care.

*Psychology of Leave.* — We have been struck with the attitude of the men under our care towards the question of leave. They don't want to be done out of their " Blighty ", and I believe it is important to make arrangements that men may have 14 days' leave given them — not sick leave, but leave to England, before they are returned to duty. Our endeavor is to get them back to duty as soon as possible, and I believe they will get well more quickly and that they will try to move their limbs more quickly if they know that they will get a fortnight's leave before going back to the line.

*Chemical Treatment.* — I believe that the treatment of wounds of war has passed out of the domain of the chemist into that of the surgeon. The important thing is that the wound should be mechanically cleansed; the multiplicity of antiseptics and chemical cleansing agents is their condemnation. It is true that some wounds cannot be mechanically cleansed and it is very desirable to have at hand a good antiseptic for use in such cases. With that end in view we are now trying a solution of Dichloramine — in

Chlorcosane, supplied to us by the Medical Research Committee. Yet in all such cases I believe the wounds must be left open to be sutured when possible secondarily.

*Bacteriology.* — Analysis of 206 consecutive reports on cultures made from wounds before operation.

| | |
|---|---|
| No growth reported . . . . . . . . . . . . . | 27 times |
| Streptococci. . . . . . . . . . . . . . . . . | 79 — |
| Anaerobes . . . . . . . . . . . . . . . . . . . . | 143 — |

Other organisms such as staphylococci and diphtheroids were also frequently present. 86.8 o/o of these wounds were therefore infected; of the streptococcal infections present — 22 were of the hemolytic variety — 57 were non-hemolytic.

Among the 123 wounds sutured primarily, 8 were infected with hemolytic streptococci, and 6 of these were complete failures.

Of the 21 failures of primary suture there were :

| | |
|---|---|
| Infected with hemolytic streptococci. . . . . . . . . . | 6 |
| " " non-hemolytic streptococci and anaerobes . | 6 |
| " " " " alone . . . . . | 4 |
| " " anaerobes alone. . . . . . . . . . . . . | 1 |
| " " staphylococci . . . . . . . . . . . . . . | 3 |
| " " aerobic bacilli . . . . . . . . . . . . . | 1 |

It would appear from this that the most dangerous organism is the hemolytic streptococcus; which statement is, I believe, in agreement with French observations.

The question now arises :

Is it essential to have a bacteriological examination made in every case before primary suture, for if so, the number of bacteriologists in the British Army would have to be increased. My own opinion is that it is not essential provided that the operator is aware of the danger of streptococci and has the time to look after the cases on which he has operated. Before delayed primary suture, when time allows, smears should be made and streptococci looked for.

5. *The Bearing of Immediate Evacuation of the Wounded upon the Question of Primary or Delayed Primary Suture.* — (1) It is obvious that the sooner the wounded can be evacuated from the front line to the operating center the better, and once efficiently dressed, so as to obviate further soiling, men should not be delayed for purpose of redressing, as no dressing or use of chemicals in the forward area can help to sterilize a wound. It is obvious also that when possible a man should be delivered to the operating centre in a fit state to be operated on at once; therefore, a system of *rechauffage* is important.

(2) After operation for primary or delayed primary suture patients should be retained for 7 days. Transit in train or car seems to cause a disturbance of the wound, resulting probably in rebleeding and exudation of serum in which organisms, that undoubtedly often remain, may find means of growing rapidly. In periods of active operations when patients cannot be retained in advanced units, the wounds should be excised as if for suture, kept moist with some innocuous fluid, and sent to a base, where they can be sutured in the course of a few days and kept.

6. *Suggestions as to Evacuation and Distribution of Wounded, with a View to Treatment of Wounds of Soft Parts.* — (1) Lightly wounded men — roughly the " walking wounded " — should not be sent to the same operating centers as the severely wounded. The " walking wounded " get away from a battle sooner than the " stretcher cases. " If they go to the same hospital as the " stretcher cases ", the natural tendency is for them to be neglected in favor of the severely wounded. Though they are many more in number, and from the military standpoint of greater value than the severely wounded, as a rule their wounds are not attended to with the same care that is extended to others.

(2) Lightly wounded men should be sent by train from the railhead clear away from the battle area to some place where they may be treated by primary suture and retained until healed. They are the first away from the battle field and, if time is not wasted, may therefore travel further. Special arrangements might have to be made for collection of records and for returns of casualties to be sent to divisional commanders. There appears to be no difficulty here that could not be overcome.

(3) If some such system as this were undertaken the number of patients on whom primary suture could be carried out would depend entirely on " medical man power. " Much, however, might be done by a careful preliminary organization for the rapid and yet efficient handling of a large number of men. A system should be introduced by which patients could be passed on with the minimum delay from reception room to undressing room, to pre-operation ward, to X-ray room, and to the operating theatre. In the theatre itself, time may be saved if each surgeon with his team works with at least 2 tables. Again, some simple and rapid form of anesthesia is desirable. Gas and oxygen anesthesia may be the best. It yet remains to be proved whether local and intraoral anesthesia may not prove of value, so as to lessen the number of anesthetists and allow them to be used instead as surgeons.

I believe that some such system as described here will be evolved so that both severely and lightly wounded men may benefit from the undoubted value of the primary suture whereby an enormous wastage of man-power may be prevented.

---

